# Metalloproteinase-Dependent TLR2 Ectodomain Shedding is Involved in Soluble Toll-Like Receptor 2 (sTLR2) Production

**DOI:** 10.1371/journal.pone.0104624

**Published:** 2014-12-22

**Authors:** Patricia Langjahr, David Díaz-Jiménez, Marjorie De la Fuente, Estefhany Rubio, Douglas Golenbock, Francisca C. Bronfman, Rodrigo Quera, María-Julieta González, Marcela A. Hermoso

**Affiliations:** 1 Disciplinary Program of Immunology, Institute of Biomedical Sciences, Faculty of Medicine, Universidad de Chile, Santiago, Chile; 2 Division of Infectious Diseases & Immunology, University of Massachusetts Medical School, Worcester, MA, United States of America; 3 Physiology Department, Millennium Nucleus in Regenerative Biology (MINREB), Faculty of Biological Sciences, Pontificia Universidad Católica de Chile, Santiago, Chile; 4 Gastroenterology Unit, Clínica Las Condes, Santiago, Chile; 5 Cell and Molecular Biology Program, Biomedical Sciences Institute, Faculty of Medicine, Universidad de Chile, Santiago, Chile; Federal University of Rio de Janeiro, Brazil

## Abstract

Toll-like receptor (TLR) 2, a type I membrane receptor that plays a key role in innate immunity, recognizes conserved molecules in pathogens, and triggering an inflammatory response. It has been associated with inflammatory and autoimmune diseases. Soluble TLR2 (sTLR2) variants have been identified in human body fluids, and the TLR2 ectodomain can negatively regulate TLR2 activation by behaving as a decoy receptor. sTLR2 generation does not involve alternative splicing mechanisms, indicating that this process might involve a post-translational modification of the full-length receptor; however, the specific mechanism has not been studied. Using CD14^+^ peripheral human monocytes and the THP-1 monocytic leukemia-derived cell line, we confirm that sTLR2 generation increases upon treatment with pro-inflammatory agents and requires a post-translational mechanism. We also find that the constitutive and ligand-induced release of sTLR2 is sensitive to pharmacological metalloproteinase activator and inhibitors leading us to conclude that metalloproteinase TLR2 shedding contributes to soluble receptor production. By expressing human TLR2 in ADAM10- or ADAM17-deficient MEF cells, we find both enzymes to be implicated in TLR2 ectodomain shedding. Moreover, using a deletion mutant of the TLR2 juxtamembrane region, we demonstrate that this domain is required for sTLR2 generation. Functional analysis suggests that sTLR2 generated by metalloproteinase activation inhibitsTLR2-induced cytokine production by this monocytic leukemia-derived cell line. The identification of the mechanisms involved in regulating the availability of soluble TLR2 ectodomain and cell surface receptors may contribute further research on TLR2-mediated processes in innate immunity and inflammatory disorders.

## Introduction

The innate immune system is essential for inducing an inflammatory response and for the activation of acquired immunity [1]. Toll-like receptors (TLRs) are a class of pattern recognition receptors (PRRs) that play a key role in innate immunity and trigger a specific immune response. TLRs are expressed predominantly in immune cells and recognize conserved structures from pathogenic (PAMPs -pathogen-associated molecular patterns-) and non-pathogenic microorganisms or endogenous ligands associated with cellular damage (DAMPs-damage associated molecular patterns-). TLRs lead to activation of transcription factors, such as NF-κB, AP-1 and IRF3, which induce the expression of cytokines, chemokines and adhesion molecules, among others. In humans, 10 TLRs have been described that recognize PAMPs/DAMPs of various chemical natures [2], [3]. TLR2 is a type I integral membrane protein that, upon recognition of PAMPs from bacteria, fungi and viruses as well as DAMPs, forms a homodimer or heterodimer with either TLR1 or TLR6 [3].

In addition to the role of TLRs in activating the immune response against pathogens, members of this receptor family have also been associated with inflammatory and autoimmune diseases [4], suggesting that TLR-signaling pathways must be tightly regulated to avoid harmful inflammatory responses [5], [6]. TLR-activation can be regulated by cytoplasmatic molecules, such as MyD88s, IRAK-M, TOLLIP and by activation of the PI3K/Akt pathway [7], [8], [9], [10]. Additionally, there is a negative regulatory function for the ectodomain of TLRs, as has been demonstrated for the soluble form of murineTLR4, a splicing variant of *tlr4* gene [11], the soluble TLR5 identified in fish [12] and soluble forms of human TLR2 (sTLR2) [13] and TLR9 [14].

Furthermore, sTLR2 has been detected in human fluids, such as plasma, breast milk, saliva and amniotic fluid as well as in supernatant of cultured monocytes [13], [15], [16]. sTLR2 functions as a regulator of TLR2 responses, playing a role as a decoy receptor and interfering with TLR2 mobilization to lipid rafts and association with co-receptor CD14 [13], [17]. In pathological conditions, such as inflammatory bowel diseases, HIV infection and acute myocardial infarction, sTLR2 levels are altered when compared to healthy subjects [18], [19], [20].

It has been suggested that sTLR2 generation involves a post-translational mechanism of the full-length receptor [13] as only one encoding TLR2 mRNA has been detected, excluding the contribution of alternative splicing [13], [21]. However, the specific post-translation mechanism for sTLR2 production has not been studied.

Proteolytic cleavage of transmembrane proteins is a common post-translational mechanism. When this process occurs at the ectodomain level, releasing a soluble fragment, it is referred as ectodomain shedding. Matrix metalloproteinases (MMPs) and disintegrinmetalloproteinases (ADAMs) are broadly studied enzymes that participate in ectodomain shedding [22], [23]. In the immune system, this mechanism is essential for generating soluble biologically active molecules, such as cytokines and their receptors, chemokines and growth factors. This process also produces a membrane-bound protein fragment which typically undergoes regulated intramembrane proteolysis (RIP), involving the γ-secretase complex [22]. In this study, we explore in monocytic cells that TLR2 proteolytic processing and sTLR2 generation triggered by Pam_3_CSK_4_, aTLR2-specific ligand. We now report that sTLR2 production, involving ADAM10- and ADAM17-dependent TLR2-ectodomain shedding contributes to soluble receptor generation in MEF cells and that the juxtamembrane domain of TLR2 is required for efficient cleavage. These results suggest that sTLR2, induced by metalloproteinase activation, functions as a negative regulator of the TLR2-induced cytokine production.

## Materials and Methods

### Ethics Statement

All clinical investigation must have been conducted according to Declaration of Helsinki principles. Participants were identified by number, not by name, and provided informed consent. The study was approved by the Institutional Review Board at Clínica Las Condes.

### Isolation of human peripheral blood CD14+ cells

Human peripheral blood CD14^+^ cells were obtained from healthy donors by negative selection using a commercially kit (RosetteSep, StemCell), following manufacturer's instructions. Briefly, EDTA was added to whole blood to a final concentration of 1 mM and 50 µL of RosetteSep Human Monocyte Enrichment Cocktail per mL; the sample was gently mixed, incubated for 20 min and then diluted with an equal volume of PBS containing 2% FBS/1 mM EDTA, followed by Ficoll-Hypaque (GE Healthcare) density-gradient centrifugation. Cells at the interface were collected, washed and plated in RPMI medium. Cell viability and CD14 content were analyzed by flow cytometry (BD FACSCalibur). Purified cells were 76–85% CD14^+^ and 91–97% viable.

### Cell culture

Isolated human peripheral blood CD14^+^ cells and the human acute monocytic leukemia-derived cell line, THP-1 (American Type Culture Collection, ATCC) were cultured in RPMI 1640 (Gibco, Invitrogen). HEK293 cells were also from ATCC and cultured in Dulbecco's modified Eagle medium (DMEM, Gibco, Invitrogen). HEK293cells stably expressing human TLR2-yellow fluorescent protein (TLR2-YFP), kindly provided by D. Golenbock [24], were cultured in DMEM medium with 0.5 mg/mL G418 (Sigma-Aldrich). Mouse embryonic fibroblast (MEF) ADAM17^-/-^ cells were provided by Amgen, USA; ADAM10^-/-^ [25] and wild type MEF by VIB Institute, Belgium and cultured in DMEM/F12 medium. All culture media were supplemented with penicillin/streptomycin (Gibco, Invitrogen), 10% heat-inactivated fetal calf serum (Gibco, Invitrogen). Cells were maintained at 37°C, 5% CO_2_.

### Chemicals reagents

The pharmacological activator and inhibitor of metalloproteinase APMA, and TAPI-1, respectively, were purchased from Biomol International. GM6001 was purchased from Chemicon International Inc., USA. PMA, cycloheximide, EDTA, DAPT and *E. coli* LPS were obtained from Sigma-Aldrich; TLR2-ligand Pam_3_CSK_4_ was purchased from Enzo Life Science. rhTLR2 was obtained from R&D Systems, USA. GI254023X was kindly provided by A. Ludwig (Universitätklinikum Aachem, Germany).

### Cell culture supernatants for sTLR2 detection

For sTLR2 analysis in conditioned media, THP-1 cells were washed with serum-free medium and 1×10^6^ cells/mL cultured in serum-free RPMI1640 medium supplemented with penicillin/streptomycin in 6-well plates. After incubation, cell supernatants were processed as described in [13]. Briefly, the supernatants were centrifuged for 5 min at 1200 rpmandfiltered (0.22 µm pore); 0.1% (v/v) Nonidet P-40 and protease inhibitors were added and concentrated 10–15 times (Centricon YM-10 concentrators; Millipore) before Western blotting or ELISA. Serial dilutions of the cell homogenates were performed and the detection of antigen was linear in the range of concentration studied.

Isolated peripheral CD14^+^ cells were incubated, for the stimulation time periods, in serum-free RPMI1640 medium; cell supernatant was collected and centrifuged at 14000 rpm for 10 min before ELISA assay.

TransientlyTLR2-YFP-transfected HEK293Tor MEF cells were incubated in serum-free medium supplemented with penicillin/streptomycin; cell supernatants were processed and concentrated 10 times as described above.

### Cell viability analysis

Cell counts and viability were determined using a hemocytometer and trypan blue dye exclusion as well as with propidium iodide exclusion method using a flow cytometry (BD FACSCalibur analyzer).

### Quantification of TLR2, IL-8, TNF-α level in cell supernatant

To eliminate contamination with cellular debris, supernatants were carefully processed by centrifugation at 14000 *rpm* and filtered through 0.22 µm pore filters. TLR2 levels were determined using a commercial enzyme-linked immunosorbent assay (ELISA) kit (DuoSet, R&D Systems). The final sTLR2 content in conditioned media was calculated, adjusting for the concentration factor.

Levels of IL-8 and TNF-α were also determined by ELISA (R&D Systems, eBioscience, respectively). All samples were analyzed in duplicate according to the manufacturer's instructions.

### Flow cytometric analysis

Specific antibodies against human TLR2, CD14 and isotype-matched control antibodies were purchased from eBioscience. For analysis of cell surface TLR2 or CD14, 3×10^5^ freshly isolated cells were suspended in PBS containing 10% human AB plasma, washed and incubated with anti-human FITC- or PE-conjugated TLR2 antibodies, anti-humanPerCP-Cy5.5 conjugated CD14 antibodies or isotype-matched antibodies for 30 min on ice. After washing, cells were fixed in 2% paraformaldehyde. Cell-surface fluorescence intensity was assessed using a FACSCalibur analyzer and CellQuest software (BD Biosciences).

### Cell transfection

TLR2-YFP (kindly provided by D. Golenbock, UMass Medical School, USA) and mutant constructs were transfected in HEK293T cells with FuGENE reagent (Roche) according to the manufacturer's protocol. Briefly, FuGENE reagent (3 µL per µg of transfected plasmid) was added to OPTIMEM (Gibco, Life Technologies) with the purified plasmid DNA and allowed to incubate for 40 min at room temperature before adding to cells plated in OPTIMEM. After 24 h, the transfection medium was replaced.

MEFs were transfected with Lipofectamine 2000 (Invitrogen, Life Technologies). The medium was replaced 6 h post-transfection. To normalize transfection efficiency in MEF cells soluble variant release levels were expressed as the ratio of sTLR2 levels in the cell culture supernatants and TLR2 in cell homogenates in ELISA assays and adjusted to the total protein content in cell homogenates by Bradford method.

### Immunoblotting

Cells were lysed with ice-cold Low Detergent Buffer (LDB) supplemented with Complete Mini Protease Inhibitor Cocktail tablets (Roche Applied Science)and disrupted by sonication three times for 10 seconds at 14 Watts on ice. The samples were centrifuged at 14000 rpm for 5 min to ensure against any cellular contamination in the supernatant. Protein concentration was determined by Bradford assay (BioRad). Equivalent amounts of total protein were separated using 10% sodium dodecyl sulphate-polyacrylamide gel electrophoresis and electrotransferred to a nitrocellulose membrane (BioRad). Non-specific binding was blocked with a solution containing 5% non-fat milk in TBS (2 mM Tris-HCl, pH 7.6, 13.7 mM NaCl) by agitation for 1 h at room temperature.

Detection of TLR2 was determined with a polyclonal anti-TLR2 (N-terminal) antibody (R&D System). Recombinant human TLR2 extracellular region (rhTLR2) was included in a lane in the gel to indicate the identity of the receptor. The specificity of the detection was confirmed by performing peptide competition assay by co-incubation of the TLR2 antibody with the rhTLR2.

Detection of TLR2-YFP was determined using an anti-GFP antibody (Abcam) followed by a goat antibody raised against rabbit IgG and conjugated to horseradish peroxidase (HRP) (Jackson ImmunoResearch Laboratories). Actin content was also detected with a specific antibody (Sigma-Aldrich). Immunoreactive bands were visualized with the enhanced chemiluminescence (ECL) Western Blotting System (Amersham).

### Negative regulation assay

THP-1 cells (5×10^5^ /well) were cultured in 500 µL of serum free RPMI medium and pre-incubated for 30 min with 100 µL of 10x concentrated medium of APMA treated or untreated-HEK293-TLR2-YFP, or mock-transfected HEK293. Cells were stimulated with Pam_3_CSK_4_ (10 ng/mL) or not (control) for 6 h, and supernatants were collected, centrifuged at 14000 rpm for 10 min and assayed for IL-8 detection by ELISA.

### Statistical analysis

Statistical analysis was performed by Student's *t*-test using GraphPad Prism 5 software. The lacks of probability are indicated.

## Results

### Production of sTLR2 by THP-1 cells occurs post-translationally

**Figure 1:**
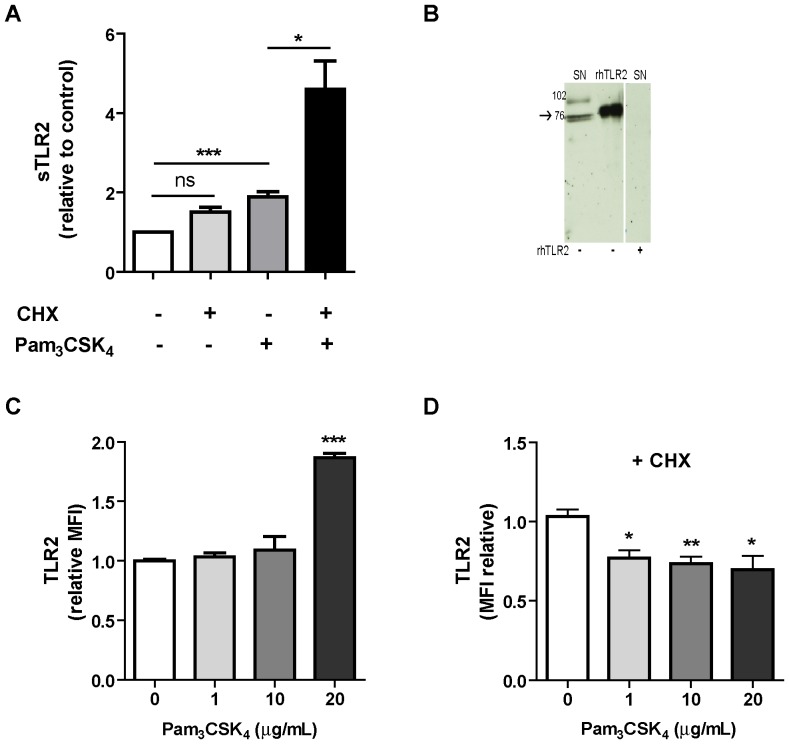
Production of sTLR2 involves a post-translation mechanism. (**A**) THP-1 cells were pretreated or not with cycloheximide (100 µg/mL) for 30 min and stimulated with Pam_3_CSK_4_ (1 µg/mL) for 5 h and the amount of sTLR2 was quantified in the cell culture supernatants by ELISA. Student t test, * p<0.05. (**B**) Detection of sTLR2 in 18 h culture supernatant (SN) of THP-1 cells by Western blotting using a N-terminal anti-TLR2 antibody. One representative of three experiments is shown. SN: cell culture supernatant; rhTLR2: recombinant human TLR2 protein. (**C**) Cell surface TLR2 levels evaluated by flow cytometry after stimulation with Pam_3_CSK_4_ (1, 10 or 20 µg/mL) for 2 h. ***, p<0.0001. (**D**) Cell surface TLR2 levels evaluated by flow cytometry after treatment with cycloheximide and then stimulation for 2 h with Pam_3_CSK_4_. MFI =  mean fluorescence intensity. *, p<0.05; **, p = 0.009.

Generation of sTLR2 can result from the post-translation modification of membrane-associated TLR2 [13]. As monocytes express high levels of membrane TLR2 and also produce sTLR2 [13], we initially examined the release of sTLR2 into culture supernatant of the acute monocytic leukemia-derived cell line, THP-1, by ELISA. Here, we show that constitutive sTLR2 levels did not decrease, but ligand-stimulated sTLR2 indeed increased in the presence of the translation inhibitor cycloheximide (Fig. 1A). More than 90% of the cells excluded trypan blue, eliminating the possibility that the treatment induced cell death. Concordant with Dulay, A. et al. [16], sTLR2 levels in culture supernatant increased with cycloheximide treatment. This result reveals that sTLR2 production involves a post-translation mechanism that may not require alternative splicing.

The presence of sTLR2 in THP-1 culture supernatant was determined by Westernblot analysis, using an anti-TLR2 ectodomain antibody, which revealed two major specific bands of ∼73–75 kDa, with a migration pattern similar to that of recombinant TLR2 (rhTLR2) in reducing conditions (75–80 kDa) (Fig. 1B), suggesting that these bands correspond to the TLR2 ectodomain. Although additional experiments are required to reach a final conclusion, the two immunoreactive bands can correspond to glycosylated sTLR2 polypeptides with slightly different electrophoretic mobility, as was previously described [26]. The ∼102 kDa TLR2 band corresponds to the full-length receptor, consistent with the molecular weight previously reported for the full-size TLR2 glycoprotein [27]. Similar results for the full-length TLR2 were also previously obtained by using freshly isolated monocytes lysates [13].

Addition of Pam_3_CSK_4_ alone augmented the levels of membrane-associated TLR2 (Fig. 1C), as was previously reported [28]. To understand the post-translational mechanism involved in sTLR2 production, the levels of membrane-associated TLR2 were determined in Pam_3_CSK_4_–stimulated cells that have been pre-treated with cycloheximide. A significant decrease in membrane-associated TLR2 was observed after 2 h (Fig. 1D).

These results demonstrate that in the absence of *de novo* protein synthesis, receptor level decreases in plasma membrane. This effect may be due to TLR2 shedding, although these data do not rule out other mechanisms that could reduce surface receptor levels, such as endocytosis. However, our observations suggest the occurrence of membrane TLR2 decrease and TLR2 *de novo* synthesis, simultaneously.

Then, the effect of pro- and anti-inflammatory molecules on sTLR2 production was studied. Cell treatment with phorbol ester (PMA) or Pam_3_CSK_4_, known activators of metalloproteinases [29], [30], induced and increased in sTLR2 production in a time-dependent manner (S1A Fig.), which is consistent with previous reports [13]. sTLR2 was constitutively produced by THP-1. Pam_3_CSK_4_ also increased sTLR2 production from isolated human peripheral CD14^+^ cells (S1B Fig.). Moreover, when THP-1cells were stimulated with LPS, a TLR4 ligand, production of sTLR2 also increased (S1C Fig.), suggesting that broad pro-inflammatory stimuli promote production of the soluble receptor.

When levels of membrane-associated TLR2 were determined in PMA-treated THP-1 cells, a significant decrease was seen, even after a short stimulation period of 30 min, and this decrease was maintained up to 120 min (S1D Fig.). Reduced membrane TLR2 levels in cells exposed to PMA treatment may be produced by a TLR2 metalloproteinase shedding mechanism. In contrast, Pam_3_CSK_4_ treatment increases TLR2 levels on the cell surface, confirming the previously described observations by Li, CH et al. [28]. These results suggest that different mechanisms occur simultaneously: TLR2 ectodomain shedding, and *de novo* expression of TLR2. PMA, compared to TLR2 agonists, is widely known for its strong effect on activation of several intracellular signaling pathways that converge in the induction of metalloproteinases.

Based on previous results indicating that the anti-inflammatory agent dexamethasone did not counteract Pam_3_CSK_4_-induced TNF-α expression [10], sTLR2 production was analyzed in cells exposed to both stimuli. We found that dexamethasone did not decrease ligand-inducedsTLR2 generation (S1E Fig.).

### Constitutive and ligand-induced sTLR2 production involves metalloproteinase-dependent TLR2shedding in human monocytes

**Figure 2:**
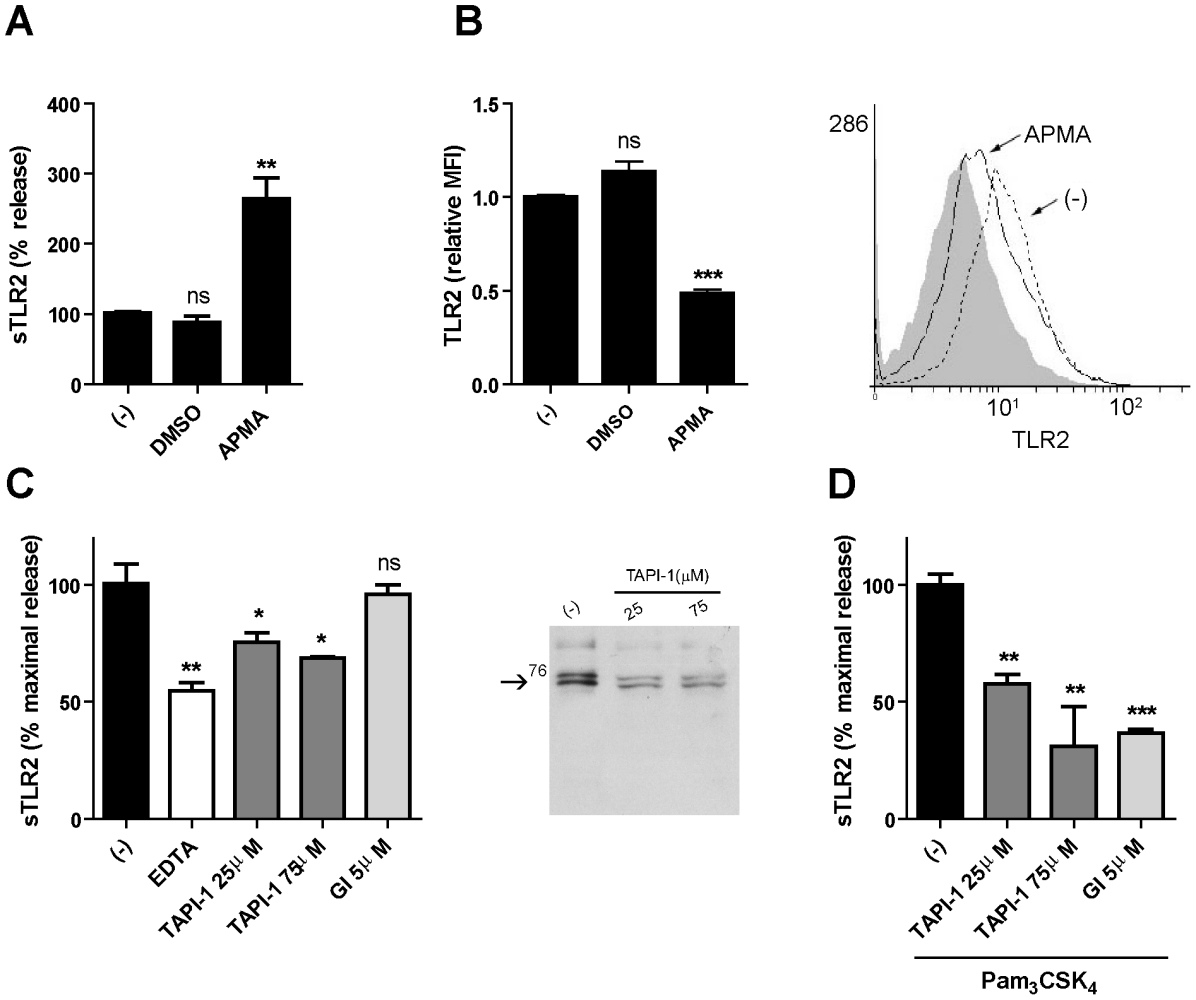
Metalloproteinase activity regulates sTLR2 release and TLR2 surface content from THP-1 cells. (**A**) Cells were treated with APMA (10 µM), vehicle (DMSO) or left untreated (-) for 5 h and the concentration of sTLR2 in the supernatants was determined by ELISA. Student t-test, **, p = 0.003. (**B**) Cell surface TLR2 was examined on these cells after 2 h addition of APMA (10 µM), vehicle (DMSO) or left untreated (-) using anti-TLR2-FITC conjugated antibody and analysis by flow cytometry. ***, p<0.0001. MFI =  mean fluorescence intensity. A representative histogram is shown using cells stained with isotype control antibody (filled histogram), untreated cells (dot line) and APMA-treated cells (black line). (**C**) Cells were treated for 18 h with EDTA (2 mM), TAPI-1 (25 µM or 75 µM), GI254023X (5 µM) (GI) or left untreated (-) and the sTLR2 concentration in the cell supernatants was determined by ELISA. *, p<0.05; **, p = 0.003. Westernblot (10% SDS-PAGE reducing gel) shows sTLR2 release in cell supernatant of TAPI-1 or left untreated (-) treated THP-1 cells using N-terminal anti-TLR2 antibody. One representative of three experiments is shown. (**D**) Cells treated with TAPI-1 (25 µM or 75 µM), GI254023X (GI) (5 µM) or left untreated (-) were added 30 min prior to cell stimulation with Pam_3_CSK_4_ (1 µg/mL) for 18 h and the concentration of sTLR2 in cell supernatants was assayed by ELISA. Data represent the mean ± SE of three independent determinations. Student t test **, p = 0.001; ***, p = 0.0001.

Ectodomain shedding of immune-related molecules, such as TNF-α and its receptors, occurs from the surface of leukocytes by the effect of metalloproteinases [31]. As shown above, PMA and Pam_3_CSK_4_ increase sTLR2 levels. Both agents are known to induce metalloproteinase activation [32], [33]. To test whether or not metalloproteinases are involved in constitutive and ligand-induced sTLR2 production in THP-1 cells, we stimulated with p-aminophenylmercury acetate (APMA), a broad metalloproteinase activator. We found that APMA promoted an increase in sTLR2 concentration (Fig. 2A) and, as expected, a decrease in membrane-associated TLR2 levels (Fig. 2B and right histogram), with no changes in viable cell count (data not shown). Similarly, shedding of TNF-α was also observed in cells stimulated with APMA, reflected by its increased level in cell culture supernatants, as well as by its decreased content in the cell surface (data not shown). These results suggest that a proteolytic cleavage of membrane-associated TLR2 could be implicated in the mechanism of sTLR2 generation.

To confirm the participation of a metalloproteinase, pharmacological inhibition of constitutive sTLR2 production was assessed initially using EDTA (2 mM), a chelator of divalent ions, necessary for metalloproteinase cleavage. A significant decrease in sTLR2 concentration in the supernatant was observed (Fig. 2C), suggestive that MMPs may be participating in TLR2 shedding, thus supporting the use of other pharmacological strategies. Accordingly, similar results were obtained with the broad-spectrum metalloproteinase inhibitor, TAPI-1, showing a decrease in sTLR2 content (Fig. 2C). An inhibition curve of basal sTLR2 release was made with TAPI-1, and we use the concentration where the cells were metabolically active and not undergoing cell death (less than 5% tripan-blue positive cells in the culture) (data not shown). These results indicate that metalloproteinase activity can contribute to constitutive sTLR2 generation, however they cannot rule out that other mechanisms may be participating in the soluble receptor production.

Treatment with TAPI-1 shown a diminution in sTLR2 production (Fig. 2C) as detected by western blotting using an N-terminal anti-TLR2 antibody. The two bands of ∼73 and 75 kDa have similar molecular size to the recombinant TLR2 ectodomain (see also Fig. 1B). A less intense TLR2 immunoreactive band of ∼100 kDa was also observed that might correspond to the full-length TLR2 [27] associated to membrane vesicles.

To define whether sTLR2 release by metalloproteinase activity is a ligand-dependent mechanism, we determined the effect of TAPI-1 on cells challenged with Pam_3_CSK_4_ and a significantly reduction of sTLR2 production was observed (Fig. 2D), with no changes in viable cell count (data not shown). Similarly, TAPI-1 inhibits constitutive and TLR2-mediated production of TNF-α, reflected by decreased levels in cell culture supernatants (data not shown). These results confirm the involvement of members of the metalloproteinase subfamily in the TLR2-driven sTLR2 generation. Despite the fact that metalloproteinase shedding participates in sTLR2 production, we cannot rule out other complementary mechanism for its generation in particular considering that TAPI-1 inhibited sTLR2 production in resting cells by ∼30%.

In order to determine whether a specific metalloproteinase could be implicated in TLR2 shedding, we used a pharmacological inhibitor with selectivity for ADAM10, GI254023X [34]. No effect of GI254023X was observed on constitutive sTLR2 production (Fig. 2C); in contrast, decreased Pam_3_CSK_4_-induced sTLR2 content was observed (Fig. 2D), no change in viable cell count was observed (data not shown). This result demonstrates that TLR2 cleavage induced by the receptor ligand was dependent on the activity of ADAM10 in THP-1 cells; although this observation cannot exclude the involvement of other metalloproteinases.

**Figure 3:**
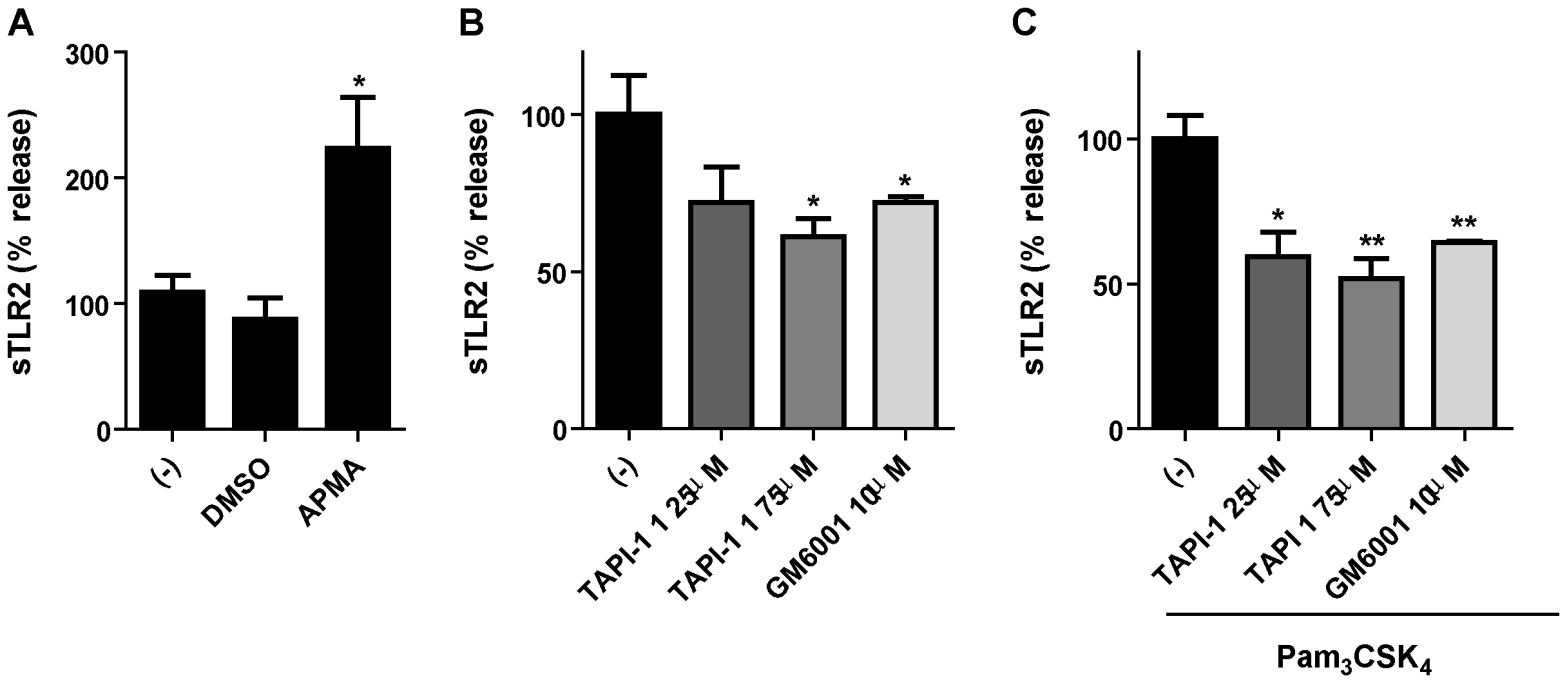
TLR2 shedding is involved in sTLR2 generation in human peripheral monocytes. (**A**) Isolated human peripheral CD14^+^ cells were treated with APMA (10 µM), vehicle (DMSO) or left untreated (-) for 5 h. *, p<0.05. (**B**) Cells were incubated for 18 h with TAPI-1 (25 µM or 75 µM), GM6001 (10 µM) or left untreated (-).*, p<0.05.(**C**) Cells treated with TAPI-1 (25 µM or 75 µM), GI254023X (GI) (5 µM) or left untreated (-) were added 30 min prior to cell stimulation with Pam_3_CSK_4_ (1 µg/mL) for 18 h. Supernatants were harvested and soluble receptor was measured by ELISA. *, p<0.05; ** p<0.01. Data are express as percentage of maximal release ± SE of two independent determinations using four different healthy donors.

To confirm that constitutive and ligand-induced sTLR2 production involves metalloproteinase-dependent TLR2 shedding in monocytes, isolated human peripheral CD14^+^ cells were stimulated with APMA or pharmacological metalloproteinase inhibitors. TNF-α levels in supernatant were also quantified by ELISA. TAPI-1 and GM6001 inhibit constitutive and TLR2-mediated production of TNF-α (data not shown). Accordingly to assays with THP-1 cell line, metalloproteinase activator increase sTLR2 production (Fig. 3A). Metalloproteinase inhibitors, TAPI-1 and GM6001, decreased constitutive (Fig. 3B) and Pam_3_CSK_4_-induced (Fig. 3C) sTLR2 generation, confirming that TLR2 shedding is involved in soluble receptor generation in human monocytes.

### ADAM10- and ADAM17-mediated TLR2 shedding

**Figure 4:**
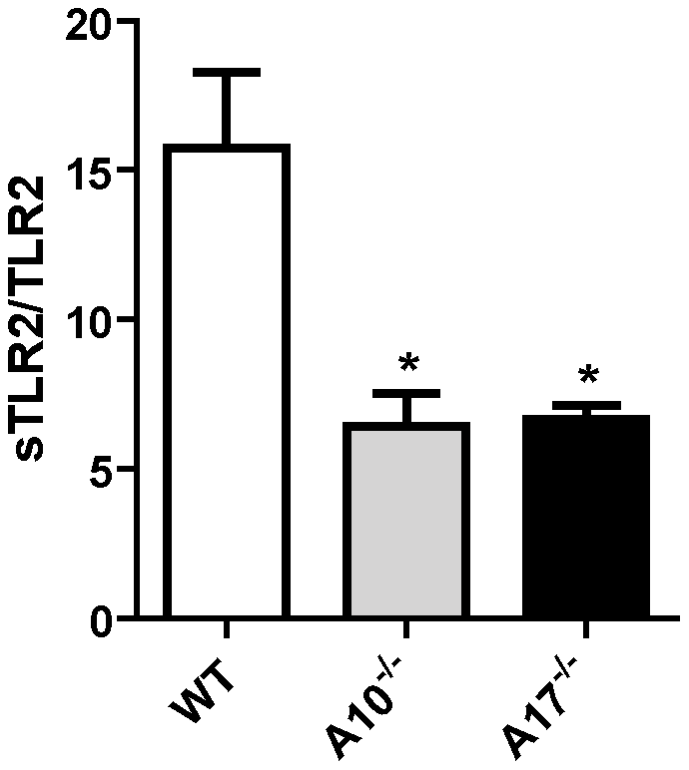
ADAM10 and ADAM17 are involved in TLR2 shedding. MEF cells were transfected with an expression plasmid encoding human TLR2 or with an empty vector; sTLR2 content in cell supernatants was analyzed by ELISA. sTLR2 content (pg/mL) was normalized to total TLR2 cell levels (ng/mg total protein). Data represent the mean ± SE of three independent determinations. *, p<0.05.

The participation of ADAM10 and ADAM17 in TLR2 ectodomain shedding was further investigated using MEFs generated from ADAM10- and ADAM17-deficient mice. Release of sTLR2 was significantly reduced in ADAM10- and ADAM17-deficient MEF cells transiently expressing human TLR2 (Fig. 4), demonstrating that these two enzymes can contribute to TLR2 shedding. If this is the case, i.e. that both ADAM 10 and ADAM17 are involved in TLR2 shedding, our results suggest that, in the absence of one of them, the other compensate and induce TLR2 shedding. Several studies have shown that in the absence of one enzyme in deficient-cell or animal models, a different, non-canonical enzyme can substitute for its function and act on the substrate [35]. Therefore, our studies cannot exclude other enzymes and/or other mechanisms participating in sTLR2 generation in MEF cells.

The content of sTLR2 in conditioned media of TLR2-transfected MEF cells stimulated with Pam_3_CSK_4_ was not detected. Indeed, over 60% of cell did not survive this treatment as detected by trypan-blue exclusion (data not shown). This result could be due to a greater susceptibility of TLR2-expressing ADAM10^-/-^ or ADAM17^-/-^ MEFs cells to undergo apoptosis when exposed to Pam_3_CSK_4_. As reported previously, Pam_3_CSK_4_ activates a cellular death pathway via TLR2, and probably the lack of function of a shedding mechanism might impact on sTLR2 generation controlling TLR2-induced apoptosis [36].

### Deletion in TLR2 juxtamembrane region impairs sTLR2 production

**Figure 5:**
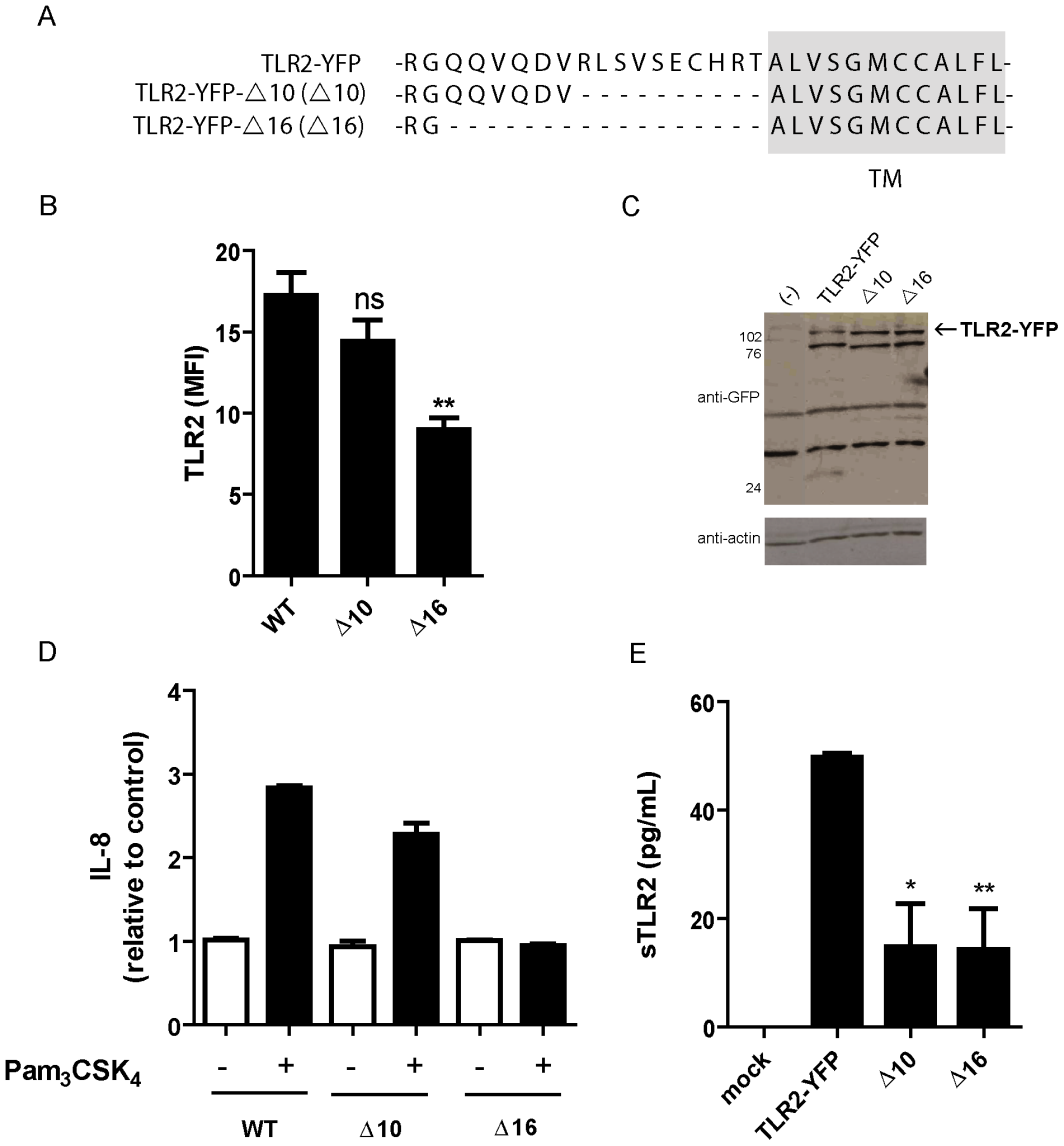
Deletion of TLR2 juxtamembrane region impairs sTLR2 generation. (**A**)Amino acid sequence of wild type (TLR2-YFP) surrounding the potential TLR2 transmembrane domain and deletion mutants, lacking 10 (TLR2-YFP-Δ10) or 16 (TLR2-YFP-Δ16) amino acids. Student t test, ** p<0.01. (**B**) HEK293T cells were transiently transfected with TLR2-YFP, TLR2-YFP-Δ10 or TLR2-YFP-Δ16, and TLR2 surface expression was evaluated by flow cytometry with anti-TLR2-PE antibody. MFI =  mean fluorescence intensity. Student t test, ** p<0.01. (**C**) Westernblot analysis, using anti-GFP antibody, of transfected cell lysates described in (B) Mock transfected HEK-cells (-); bands correspond to two GFP immunoreactive TLR2-YFP polypeptides. (**D**) Pam_3_CSK_4_-induced IL-8 production by wild type and deletion mutant expressing cells was evaluated by ELISA. (**E**) sTLR2 content in cell supernatants, relative to Pam_3_CSK_4_-induced wild type cells, of wild type or Δ10 or Δ16 deletion mutant transfected cells was evaluated by ELISA. * p<0.05; ** p<0.01. All graphs show mean values ± SE of three independent experiments.

Although there is no consensus amino acid sequence for proteolytic cleavage by metalloproteinases; most transmembrane proteins that are susceptible to metalloproteinase shedding are cleaved at the juxtamembrane region [23]. To evaluate the potential cleavage site of metalloproteinase(s) in TLR2, we generated two juxtamembrane-region deletion-mutants, of 10 (TLR2-YFP-Δ10) and 16 (TLR2-YFP-Δ16) amino acids, respectively, corresponding to the sequence between the last TLR2 leucine-rich-repeat (LRR) motif and the transmembrane region (Fig. 5A). Control (TLR2-YFP) and mutant constructs were transiently transfected into HEK293T cells that do not endogenously express TLR2. The TLR2 surface content, evaluated by flow cytometry, was similar inTLR2-YFP and TLR2-YFP-Δ10, but was significantly decreased in TLR2-YFP-Δ16 (Fig. 5B), even though theTLR2 content in whole cell lysates of the mutants was similar (Fig. 5C). The ∼110 kDa band in the Westernblot corresponds to TLR2-YFP, which is absent in the lysate of TLR2-YFP (-) cells. The ∼80 kDa band could correspond to a fusion protein (TLR2-YFP), albeit with a different degree of glycosylation. Whereas YFP is fused to the TLR2 C-terminus, the ∼80 kDa band does not correspond to the TLR2 ectodomain of closest electrophoretic mobility (see Fig. 1B), because upon MMPs shedding it lacks the YFP-tag.

These findings suggests that removal of 16 amino acids from the TLR2 juxtamembrane region affects TLR2 cell membrane expression, as has been shown for juxtamembrane region deletion mutants ofMHC Class I-related chain A (MICA) [37]. To confirm that the mutation affects membrane-anchored receptor function, we found no increased production of IL-8 in TLR2-YFP-Δ16-expressing cells stimulated with Pam_3_CSK_4_, when compared to TLR2-YFP- or TLR2-YFP-Δ10-expressing cells (Fig. 5D). This protein deletion might be affecting ligand binding to the extracellular domain that has an impact on IL-8 release (Fig. 5D). This effect can be a consequence of a conformational change in TLR2 ectodomain, leading to a decrease affinity for the ligand. However, additional studies are needed to confirm this hypothesis.

Finally, decreased sTLR2 content in cell supernatants was determined in TLR2-YFP-Δ10- and -Δ16-expressing cells in comparison to the wild type TLR2-YFP (Fig. 4E), indicating that TLR2 juxtamembrane deletion affects sTLR2 generation and that the sheddase cleavage site is located in a receptor domain close to the transmembrane region. Another possibility is that the deletion affects the structure of TLR2 ectodomain therefore is not recognized by the enzyme. The effect of Pam_3_CSK_4_ on the release of sTLR2 by Δ10 or Δ16 cells was also affected and undetectable differences with untreated cells were seen (data not shown), since the enzyme-recognition or-cleavage site in full-length TLR2 is altered in these mutants.

### sTLR2 induced by metalloproteinase-activation inhibits Pam_3_CSK_4_-mediated IL-8 production

**Figure 6:**
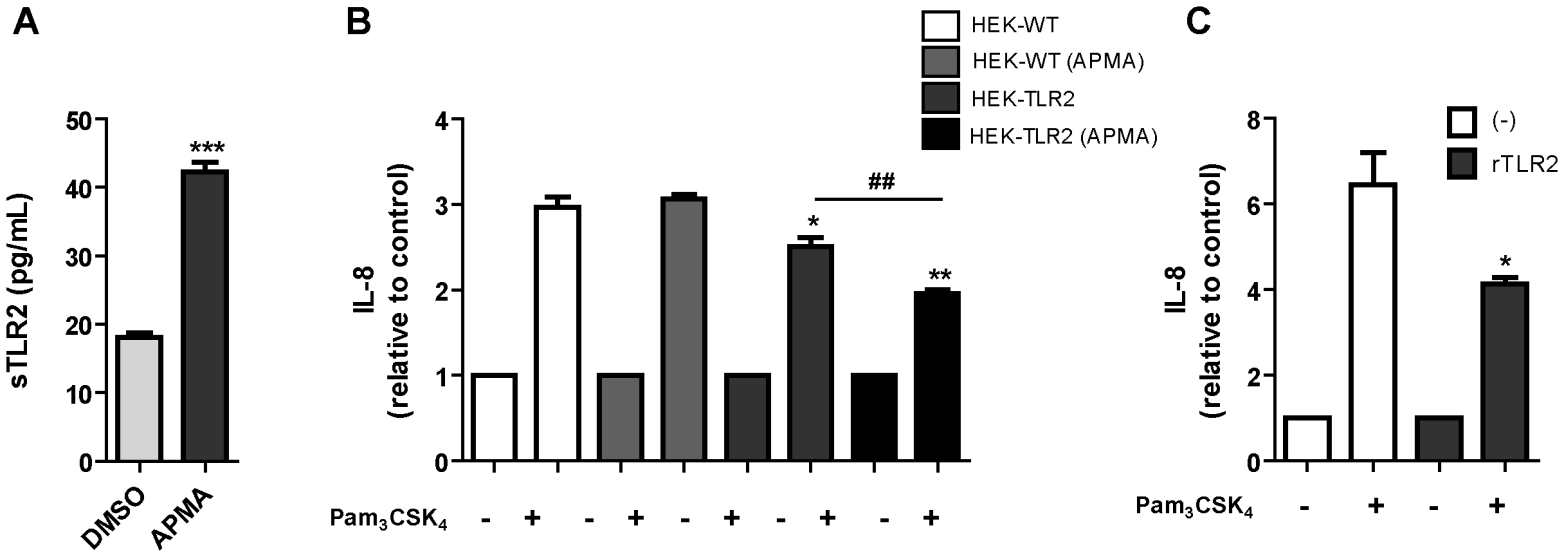
sTLR2 induced by metalloproteinase activator APMA inhibit TLR2 ligand-mediated IL-8 production. (**A**) Stably transfected HEK-TLR2-YFP cells were incubated for 24 h with fresh medium; 5 h prior to cell supernatant collections they were treated with APMA (10 µM) or DMSO; and sTLR2 content in conditioned media was determined by ELISA. Student t test ***, p<0.0001. (**B**) THP-1 cells were pre-treated with conditioned media of HEK293 (HEK-WT), APMA-treated HEK293 (HEK-WT+APMA), HEK-TLR2-YFP (HEK-TLR2) or APMA-treated HEK-TLR2-YFP cells (HEK-TLR2+APMA), and then Pam_3_CSK_4_-induced IL-8 production was determined. Student t test *, p = 0.0481; **, p = 0.0017; **^##^**, p = 0.008. (C) Pam_3_CSK_4_-induced IL-8 production by THP-1 cells, pre-treated or not with recombinant human TLR2 ectodomain. Student t test *, p<0.05. All graphs show mean values ± SE of three independent experiments.

To demonstrate the negative regulatory function of sTLR2, induced by metalloproteinase activation, we analyzed the effect of conditioned media from HEK293-TLR2-YFP cells exposed to APMA on TLR2-induced IL-8 production by THP-1 cells. Activation of metalloproteinases in HEK293-TLR2-YFPcells induced sTLR2 release (Fig. 6A), similar to the effects previously described (see Fig. 2A). IL-8 production was significantly reduced when Pam_3_CSK_4_-activated THP-1 cells were incubated with conditioned media from APMA-treated or untreatedHEK293-TLR2-YFP cells (Fig. 6B). However, inhibition of IL-8 production was higher in cells exposed to conditioned media of APMA-treated HEK293-TLR2-YFP cells compared with untreated HEK293-TLR2-YFP cells conditioned media. THP-1 cells were also incubated with conditioned media from APMA-treated or untreated HEK293, cells that do not endogenously express TLR2 and no effect on IL-8 was observed (Fig. 6B). These data suggest that sTLR2 induced by metalloproteinase activation inhibits IL-8 production, thus acting as a negative regulator. Similar results were obtained when the recombinant human TLR2 ectodomain protein was used as a control to antagonize TLR2-driven IL-8 production (Fig. 6C). Although natural sTLR2 released by cells was shown to be biologically active in negatively regulating TLR2-triggered inflammatory responses [13], the inhibition of IL-8 production by soluble variant induced by metalloproteinase activation has not been previously demonstrated. These results confirm those previously reported from natural sTLR2 and extend our knowledge about the mechanisms leading to the production of this negative regulator of innate immune and inflammatory responses.

## Discussion

In this study, we demonstrated that metalloproteinase is involved in the mechanisms that generate constitutive and ligand-induced sTLR2 and we revealed the functional role of this shed receptor on TLR2-induced cytokine production. Over-activation of TLR2 signaling is involved in multiple pathological conditions, such as autoimmune and inflammatory diseases [38]. Therefore, regulation of TLR2 activity and the signaling cascade represents an important mechanism for maintaining cell homeostasis. sTLR2 has proven to be a negative regulator of the receptor-dependent activity both *in vitro* and *in vivo*, using recombinant TLR2 ectodomain in monocyte cultures or in a mouse model of peritoneal inflammation, respectively [13], [17]. Furthermore, levels of sTLR2 are altered in pathological conditions, such as ulcerative colitis and acute myocardial infarction [18], [20]. Collectively, these data suggest that soluble variants of TLR2 play an important role in regulating the receptor activation and in mechanisms leading to inflammatory diseases. Thus, how these pro-inflammatory conditions impact on the mechanisms by which sTLR2 are generated is of vast interest.

The study confirms sTLR2 production is increased by activation of peripheral monocytes and a monocytic-leukemia derived cell line with Pam_3_CSK_4_ suggesting that TLR2 activation increases its own shedding. PMA and LPS, other potent inflammatory stimuli, also increased production of the soluble receptor, presumably because they converge at some level in the signaling pathway. Moreover, the broadly used anti-inflammatory agent dexamethasone did not decrease the ligand-induced sTLR2 generation, suggesting that the intracellular events, perhaps including the PI3K pathway, triggered by TLR2 activation in the presence of glucocorticoid [10], are also involved in this process.

The mechanism contributing to sTLR2 generation has not yet been completely clarified. Inhibition of protein translation did not decrease sTLR2, demonstrating that the mechanism involved in sTLR2 generation is a post-translation process and does not require alternative splicing, as was previously shown [13]. Strikingly, cycloheximide treatment lead to an increased in sTLR2 production in cells stimulated with Pam_3_CSK_4_. Cells exposed to cycloheximide are subjected to translation inhibition that may decrease the amount of many regulatory proteins involved in sTLR2 production and/or processing (such as a TIMP-tissue inhibitor of metalloproteinase-), resulting in an increased processing of the membrane-anchored TLR2. In this regard, the translation inhibitor cycloheximide inhibited stimulated TIMP-1 production in human astrocytes or mouse osteoblastic cells [39], [40] and basal and stimulated TIMP-1 and TIMP-2 activity [41], [42]. It is noteworthy that ADAMs activity is regulated by multiple mechanisms [35], not only by TIMPs, cannot be ruled out that these regulatory mechanisms are also affected by the translation inhibitor.

Additionally, cycloheximide treatment in TLR-activated THP-1 cells leads to a super-induction of IL-1 gene transcription. This is due to inhibition of re-synthesis of NF-κB repressor IκB-α. IκB-α binds to the activated NF-κB in the nucleus resulting in the inhibition of NF-κB activity [43] and this transcription factor participates in metalloproteinase activity regulation.

Well-known activators of metalloproteinase, such as PMA and APMA [32], decreased the content of TLR2 in the plasma membrane and increased sTLR2. These data led us to evaluate whether TLR2 is subjected to protein ectodomain shedding. This post-translation mechanism is highly conserved for membrane-associated proteins of the immune system, which regulates the availability of many molecules, such as cytokines and chemokines, among others [44].

Since production of sTLR2 is sensitive to metalloproteinase inhibitors, in particular TAPI-1 and GI254023X and induced a decrease in soluble receptor by activated cells, we suggest that sTLR2 production by metalloproteinase shedding is higher in activated cells. Similar results were observed with the production of the soluble variant of the TREM-1 receptor, where the metalloproteinase inhibitor GM6001affects its release in LPS-stimulated monocytes [45].

As TLR2 shedding was inhibited by TAPI-1 [46], a metzincin metalloproteinase inhibitor, the TLR2 sheddase(s) might be a member of this enzyme subfamily. Shedding was also diminished by GI254023X, a preferential inhibitor of ADAM10, indicating an involvement of this metalloproteinase in ligand-induced sTLR2 generation in human monocytes. ADAM10 participates in the constitutive shedding of many substrates; such as transmembrane chemokines and adhesion molecules [47], Notch [48] and low-affinity immunoglobulin E receptor CD23 [49]. Our results show that ADAM10 cleaves TLR2 in Pam_3_CSK_4_-activated monocytes, establishing ADAM10 as an inducible sheddase for TLR2, as has been also reported for IL-6R [50].

Indeed, we find sTLR2 production is decreased in both ADAM10^-/-^ or ADAM17^-/-^ MEFs expressing human TLR2, suggesting that both enzymes contribute to human TLR2 shedding in murine cells. However, in the THP-1 cell line preferential inhibition of ADAM10 does not affect sTLR2 constitutive production, which could be ascribed to a difference in the enzymes involved in TLR2 proteolytic processing in different species, such us IL-6R shedding [50]. ADAM10 and ADAM17 are important regulators of the immune system as they are responsible for ectodomain shedding of several proteins, such as TNF-α [51] and CX3CL1 [47]. Many substrates are processed by both enzymes; however, other proteins are processed by either ADAM10 or ADAM17 [52]. Our results let us to conclude that TLR2 fits in the class of substrates processed by both proteases.

In the context of the innate immune response, shedding of TLR2 represents a strategy by which a cell can down regulate the immune response triggered by a microorganism or alarmins sensed by an antigen-presenting cell, a monocyte or dendritic cell. By increasing TLR2 levels and a simultaneously decrease in membrane TLR2 availability, an excessive inflammatory response might be avoided. Other members of the IL1-R/TLR superfamily undergo ectodomain shedding, such as IL-1R2 [53] and TLR9 [14]. Additionally, TLR9 is also subjected to N-terminal domain cleavage to promote receptor activation [54]. Collectively, our data and the other reported information suggest that proteolytic processing of TLR contribute to regulate receptor signaling.

Due to an up-regulation of metalloproteinases in many inflammatory disorders, high levels of sTLR2 might be produced to avoid detrimental responses. We found that sTLR2 generated by metalloproteinase activation inhibited Pam_3_CSK_4_-mediated IL-8 production by monocytes, suggesting that the TLR2-shedding product is a functional negative regulator of the receptor pathway and proteolytic processing of the receptor downregulates inflammatory signaling.

In summary, the identification of TLR2 metalloproteinase shedding as a significant component involved in soluble receptor generation is an important contribution to the understanding of TLR2 signaling regulation and sTLR2 pathophysiological function.

## Supporting Information

S1 Fig **sTLR2 production by cells stimulated with different pro- and anti-inflammatory molecules.** (**A**)THP-1 cells were treated or not with Pam_3_CSK_4_ (1 µg/mL), PMA (50 ng/mL) for 5 or 18 h and then the amount of sTLR2 quantified in the cell culture supernatant by ELISA. Student t test *, p<0.05; ***, p = 0.0007. (**B**) Isolated peripheral CD14^+^ cells were stimulated with Pam_3_CSK_4_ (1 µg/mL) for 18 h. Student t test *, p<0.05. (**C**) THP-1 cells were treated or not with LPS (1 µg/mL) for 18 h and sTLR2 content in the cell culture supernatant quantified by ELISA. Student t test *, p<0.05. (**D**) Surface TLR2 levels after treatment of THP-1 PMA (50 ng/mL) for the indicated times. Student t test **, p<0.01. (**E**) THP-1 cells were pre-treated with dexamethasone (10 nM) and stimulated with Pam_3_CSK_4_ (1 µg/mL) for 18 hand sTLR2 content in the cell culture supernatant quantified by ELISA.
(TIF)
