# Supplementary figures and images for: Metalloproteinase-Dependent TLR2 Ectodomain Shedding is Involved in Soluble Toll-Like Receptor 2 (sTLR2) Production
Source: PLoS One. 2014 Dec 22;9(12):e104624. doi: 10.1371/journal.pone.0104624 (PMC4273945; doi:10.1371/journal.pone.0104624)

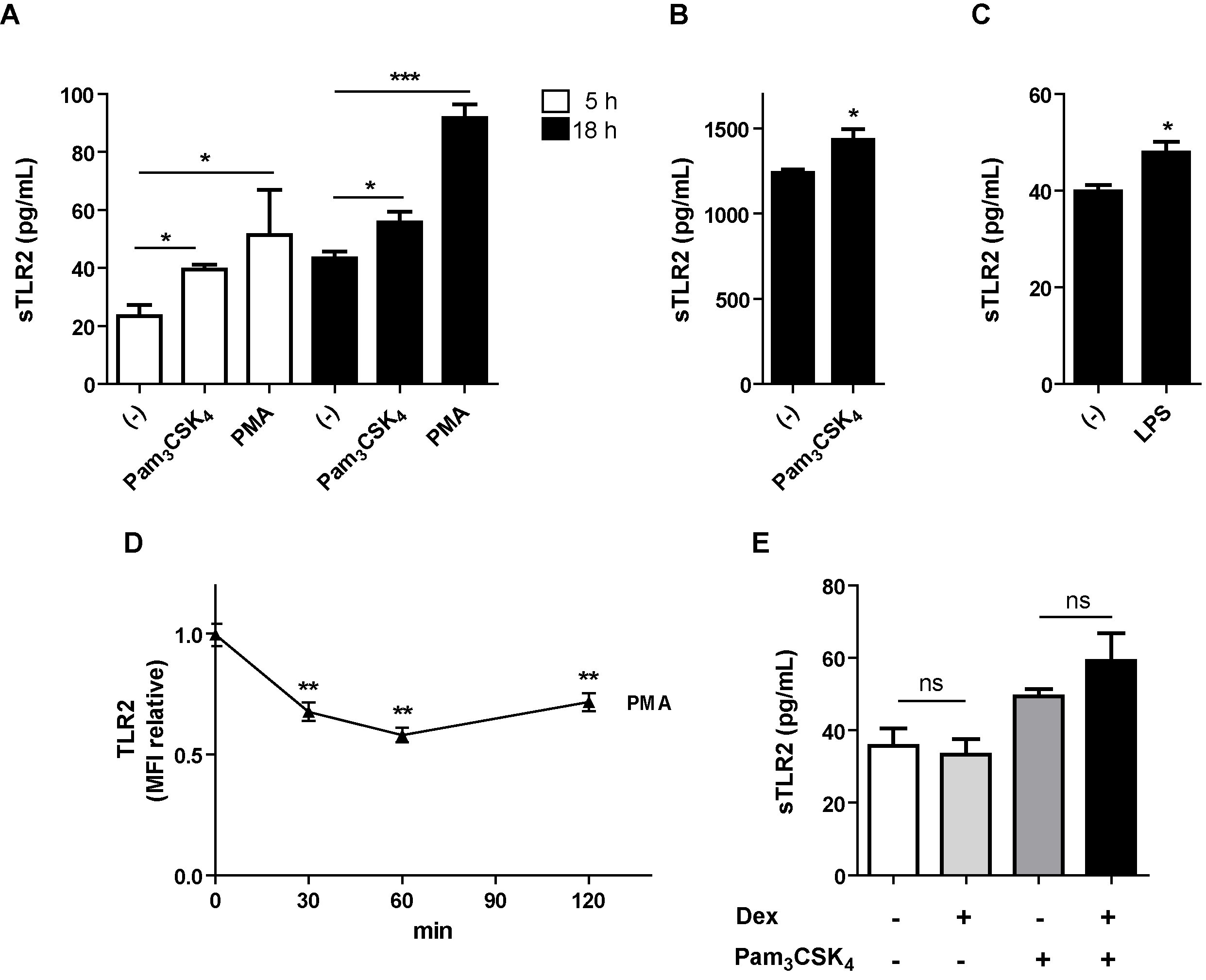

Supplement: S1 Fig — sTLR2 production by cells stimulated with different pro- and anti-inflammatory molecules. (A)THP-1 cells were treated or not with Pam3CSK4 (1 µg/mL), PMA (50 ng/mL) for 5 or 18 h and then the amount of sTLR2 quantified in the cell culture supernatant by ELISA. Student t test *, p<0.05; ***, p = 0.0007. (B) Isolated peripheral CD14+ cells were stimulated with Pam3CSK4 (1 µg/mL) for 18 h. Student t test *, p<0.05. (C) THP-1 cells were treated or not with LPS (1 µg/mL) for 18 h and sTLR2 content in the cell culture supernatant quantified by ELISA. Student t test *, p<0.05. (D) Surface TLR2 levels after treatment of THP-1 PMA (50 ng/mL) for the indicated times. Student t test **, p<0.01. (E) THP-1 cells were pre-treated with dexamethasone (10 nM) and stimulated with Pam3CSK4 (1 µg/mL) for 18 hand sTLR2 content in the cell culture supernatant quantified by ELISA. (TIF) [file pone.0104624.s001.tif]
